# Manganese gluconate, A greener and more degradation resistant agent for H_2_S oxidation using liquid redox sulfur recovery process

**DOI:** 10.1016/j.heliyon.2020.e03358

**Published:** 2020-02-10

**Authors:** Tirto Prakoso, Andreas Widodo, Antonius Indarto, Rina Mariyana, Aditya Farhan Arif, Tri Partono Adhi, Tatang Hernas Soerawidjaja

**Affiliations:** a Department of Chemical Engineering, Institut Teknologi Bandung, Labtek X, Kampus ITB, Jalan Ganesha 10, Bandung, 40132, Indonesia; b PT. Energy Management Indonesia (EMI), Jl. Pancoran Indah I No. 52, Jakarta, Indonesia; c PT. Rekayasa Industri (REKIND), Jl. Kalibata Timur I No. 36, Jakarta, Indonesia

**Keywords:** Chemical engineering, Environmental chemical engineering, Industrial chemistry, Environmental chemistry, Environmental hazard, Manganese gluconate, Sulfur recovery, Liquid redox, Natural gas, Acid gas, Elemental sulfur

## Abstract

Iron chelate liquid redox sulfur recovery (LRSR) has been one of the most frequently recommended technologies for the oxidation of H_2_S in natural gas into elemental sulfur, particularly when the acid gas has a high CO_2_/H_2_S molar ratio. The process is however known to suffer from extensive oxidative ligand degradation that results in high operational costs. Moreover, poor biodegradability or toxicity of the existing ligand has become a concern. In this research, we demonstrated that gluconate, a naturally greener ligand, when coupled with manganese as the metal, has considerable potential to be a better redox agent. Manganese gluconate solution was more resistant against ligand degradation compared with iron NTA. As required, aerated solution was capable of converting dissolved NaHS into elemental sulfur. At sufficiently high pH, manganese gluconate solutions were stable enough from precipitation of manganese hydroxide, carbonate, or sulfides. An equilibrium calculation has been developed to understand the precipitation behavior.

## 1 Introduction

Hydrogen sulfide and carbon dioxide are common impurities of natural gas. Due to process, transportation, and safety concerns, these components have to be removed. The separated acid gases cannot be just disposed freely because of the hazardous nature of H_2_S to humans and the environment. The substance has to be captured and generally converted into elemental sulfur. Recovery of sulfur using common industrial methods, such as Claus or Selectox process, will likely give operational severe problems when the molar ratio of CO_2_ to H_2_S in the acid gas is very high (CO_2_/H_2_S > 33) [1, 2]. The situation worsened if the gas also contains a significant quantity of heavy hydrocarbons particularly BTEX (Benzene, Toluene, Ethylbenzene, and Xylene). For such cases, one of the recommended technologies is the iron chelate Liquid Redox Sulfur Recovery (LRSR) process [3]. The technology is well known for its high sulfur recovery efficiency (>99.9%), simple operation, and higher tolerance toward BTEX.

High circulation rate of solvent and chemical make-up bring about high capital and operating costs of iron chelate LRSR process [3, 4, 5]. The technology is usually attractive only for small sulfur production capacity, i.e., 1–3 ton/day [6] and becomes economically prohibitive when sulfur rate is greater than 20 metric tons/day [7]. The high operating costs have been caused by rapid oxidative degradation of the ligands such as EDTA (Ethylenediaminetetraacetic acid), HEDTA (2-hydroxyethylenediaminetriacetic acid), and NTA (Nitrilotriacetic acid) [4, 5, 8]. Chen et al., for instance, reported that at pH between 7 and 8.5, half of the initial NTA has already degraded only within 25–40 h [10]. The destruction which decomposes ligands into smaller subtances ineffective for complexing the metal has been attributed to Fenton [9] or Ruff mechanisms [10]. Several investigations have been carried out to overcome this problem. One of these was the introduction of citric acid as an additional ligand to Fe-EDTA, which improved the degradation of EDTA. The method however was reported to have caused deterioration of sulfur recovery performance [5] Other works were the development of new ligands, which currently still resulted in ligands that have either unsatisfying oxidative degradation rate or low complex stability against metal salt precipitation [4]. Moreover, production of the new ligands may be too costly [4]. In addition to oxidative degradation resistance, LRSR complex stability mentioned above is also very important; even if a ligand was not degraded by oxidation at all, but if the complex it forms is not stable to metal precipitation then the sulfur recovery will be low. Other disadvantages of conventional ligands are that the biodegradability of the EDTA is weak, and the use of NTA should be restricted because of its toxicity [11].

Novel liquid oxidation processes have been developed to avoid the weaknesses of the iron chelate process. Gendel et al. [12] proposed a technique using a low pH condition of iron LRSR equipped with electrochemical regeneration. The process did not require the use of any ligands. Unfortunately, it still gave low recovery efficiencies, i.e. not higher than 80%. Biological desulfurization is another type of process which has been applied commercially. However, growing the microbes on the industrial scale is not easy [13]. From industrial experience it appears that the bacteria may be deactivated by the presence of BTEX. H_2_S capture and conversion using ionic liquids that contain redox agents have also been reported [8, 13]. In the work of Guo et al., N,Ndimethylformamide (DMF) was added to reduce the high viscosity of the ionic liquid [13]. The process seems to be very promising because it shows high recovery efficiency, it does not use any ligand and has no over-oxidation products. Nevertheless, some fundamental issues may need to be addressed before this process can be applied industrially. These may include possible water build-up (or low-pressure dehydration would be needed), and substantial hydrocarbon absorption, which in the end may result in a high cost of ionic liquid make up [14].

The preceding development status of other LRSR technologies suggested that iron chelate technology still has advantages. Along with the fact that many operating sulfur recovery units use this technology, economic improvement without altering much of its fundamental operating principles will be preferred. The ultimate goal of whole works was to contribute to such endeavor by taking a simple approach, i.e. we did not directly put effort into synthesizing new sophisticated ligands. Instead, we tried to find out any existing environmentally friendly ligand that can reduce operating costs without degrading the technical performance of the technology. Based on the reasons to be elaborated in the next part, gluconate ligand has been selected for evaluation.

In an alkaline environment, gluconate has been reported to be superior to many chelating agents including EDTA and NTA [15, 16]. It resists oxidation and reduction even at high temperatures [17, 18]. Environmentally, sodium gluconate is a green chemical. It is biodegradable, non-toxic [19], and has been manufactured from glucose, which is produced from renewable materials. Sodium gluconate is cheaper than sodium NTA and EDTA [20, 21]. On molar basis the unit price of gluconate can be as low as one third to a quarter of the conventional ligands. It is a central strategy in this work to couple gluconate with manganese rather than iron. This is because, in contrast to the latter, manganese has been reported to slow down the degradation of several organic materials that is caused by the Fenton mechanism [22, 23, 24]. Manganese gluconate solutions have been reported to be stable, even in the presence of air [25]. However, due to the abundance of iron in nature, and for comparison purposes, iron gluconate solutions were also tested.

To the knowledge of the authors, the number of scientific publications that assess the prospect of manganese gluconate for LRSR process was limited. Deshpande et al. patented an H_2_S recovery process using metal gluconate [26]. However, they focused on iron gluconate and did not discuss the ability of manganese gluconate. Related to this limitation, this particular work therefore was aimed to make a screening stage assesment of the basic potential of manganese gluconate, before continuing with more detailed investigations. This was carried out by evaluating the following points: 1) the ability of its aerated solution to convert sulfide into elemental sulfur, 2) resistance against oxidative degradation relative to Fe-NTA, and 3) the stability of complex against metal salt precipitation. To simplify the work NaHS and Na_2_CO_3_ were used as a source of sulfide and carbonate rather than gaseous H_2_S [27] and CO_2_. Equilibrium model on metal salt precipitation was developed to help understand the experiment results.

## 2 Experimental setup

### 2.1 Materials

NaHS·xH_2_O (34% of sulfur content obtained from Sigma Aldrich, USA) was used as the source of sulfur. The sulfur content of NaHS was determined further by iodometric titration. NTA (99% purity), EDTA tetra sodium salt (97% purity), and D-gluconic acid sodium salt (99% purity) were purchased from Sigma Aldrich were used as ligands. Sodium carbonate (99.9% purity), NaOH (99% purity), MnCl_2_ 4.H_2_O (99% purity), FeCl_3_ (98% purity) and HCl were puchased from Merck Germany. Manganese (II) gluconate dihydrate (98.0–102.0% purity) and Iron (II) gluconate dihydrate (97.0–102.0% purity) were from Jost Chemical USA.

### 2.2 Solution preparation

25 ml of 0.01 M–0.1 M aqueous ferrous or manganese gluconate solutions were prepared by mixing ferrous/manganese (II) gluconate and sodium gluconate in distilled water with a magnetic stirrer in an erlenmeyer. The sodium D-gluconate was added until the desired gluconate to metal ratio of 2, 8 or 12 was achieved. Sodium hydroxide or HCl was used if necessary to adjust the pH to the intended level. Because the pH in the absorber has to be neutral or alkaline in order to effectively capture the H_2_S in the industrial application, the range of pH selected was between 7 and 13. For carbonate precipitation experiment, the solution was finally mixed with carbonate solution. The concentration and volume of the carbonate solution added were such that a final solution's concentration of 0.03–0.3 M and total volume of 50 ml was obtained. The pH could be readjusted if necessary. In sulfide precipitation experiments, instead of sodium bicarbonate sodium hydro sulfide (NaHS) was used such that the sulfide concentration in the final solutions was 10 mM.

### 2.3 Experiment for testing solution capability for converting NaHS into elemental sulfur

In the sulfur production experiment, a mixture of manganese gluconate and sodium carbonate solution at pH 13 was aerated first. After addition of NaHS, the solution was kept in the dark for few days to complete the precipitation. In order to identify the chemical species, after separation, and drying, the precipitate was analyzed using X-ray diffraction (XRD, Bruker D8 Advance, USA). The filtrate was reaerated and used for another cycle of the oxidation experiment.

### 2.4 Precipitation stability experiment

The precipitation tests for the evaluation of complex stabilities were carried out with two conditions: experiment with limited presence of oxygen carried out by nitrogen bubbling as an approach to the (lower part of) absorber condition, and experiment with air bubbling to simulate regeneration condition of an LRSR process. The bubbling was given to the distilled water for 60 min before the addition of chemicals and to the final solution along the whole experiment period (20 min).

Measurement of solution redox potential was carried out by using an oxidation-reduction potential (ORP) meter (Hanna HI98121), calibrated with the standard solution. A thermostat was employed to maintain the temperature of the solution at 50 °C at atmospheric pressure. The final solution was then observed for a maximum of 4 weeks to ensure that there was enough time for metal precipitation, if any. The precipitate was then subjected to XRD analysis. The presence of metal in the precipitate was also identified by washing of the precipitate with concentrated HCl. Huge losses due to washing indicates the presence of metal. No sodium carbonate nor sodium hydro sulfide was added to the manganese and iron gluconate solutions in the hydroxide precipitation experiments. Sulfide precipitation was also carried out for iron EDTA solution at pH 8–9 to compare the amount of sulfide in the precipitate with that of manganese gluconate system. The ratio of EDTA to iron was two and the source of iron was ferrous sulfate, while the EDTA was EDTA tetrasodium salt.

### 2.5 Oxidative degradation resistance experiment

Iron NTA solution was obtained by dissolving sequentially 0.2 mol/L NTA and 0.1 mol/L ferrous sulfate in 250 ml distilled water. Sodium hydroxide and HCl were then added to adjust pH to 8. Two manganese Gluconate solutions were prepared by dissolving 0.7 mol/L sodium gluconate in distilled water, followed by the addition of 0.1 mol/L manganese (II) chloride. The pH was then adjusted to 8 and 13. For the solution with pH of 13, sodium bicarbonate was added such that its concentration reached 0.3 M. The three solutions were then magnetically stirred and bubbled with air for 2–4 weeks for precipitation observation. The pH of the mixture was maintained by periodic adjustment with sodium hydroxide and HCl.

## 3 Equilibrium calculation

The concept of precipitation equilibrium calculation that has been developed will be described using manganese gluconate as an example. Details of the method are provided in supporting information (SI–S2). The model aims to find [*G*_*T*_]_*sat*_ which is the amount of total gluconate concentration needed to prevent solution from oversaturation for a defined system condition. The condition was determined by the solution's redox potential, pH, and also by the total manganese concentration, as well as the total sulfide or carbonate concentration. The corellation between pE and pH for different systems was obtained from the regression of measurement data, which are provided in Table 1.

**Table 1:** Linear fitting of pE of measured E (volt SHE) as a function of pH, pE = p.pH + q.

Component	Condition	p	q	R^2^
FeCO_3_	N_2_ bubbled condition	-1.5864	8.2133	0.995
FeS	N_2_ bubbled condition	-1.6623	10.433	0.999
Fe(OH)_2_	N_2_ bubbled condition	-1.6623	10.433	0.999
Fe(OH)_3_	O_2_ bubbled condition	-2.1278	18.578	0.980
MnCO_3_	N_2_ bubbled condition	-2.1225	20.389	0.992
Mn_S_	N_2_ bubbled condition	-2.1225	20.389	0.992
Mn(OH)_2_	N_2_ bubbled condition	-1.5247	14.428	0.993
MnOOH	O_2_ bubbled condition	-2.1225	20.389	0.992

The [*G*_*T*_]_*sat*_ was obtained by solving a set of equations that consist of charge and mass balances of manganese, gluconate, and carbonate, or sulfide as well as their equilibrium correlation. For simplification, it was assumed that no sulfur other than sulfides were formed. The equilibrium relations include dissociations, complex formations, and redox reactions. The dissociating species in the system were water, gluconic acid, aqueous hydrogen sulfide, bisulfite ions, carbonic acid, and bicarbonate ions. Complex assumed to form are manganese (I) gluconate, manganese (II) gluconate, manganese (III) gluconate, manganese hydroxide complexes, manganese carbonate complex, and bicarbonate complex. Due to low stabilization constants, manganese sulfide complex formation was neglected. Redox equilibrium taken into account was the equilibrium between Mn^3+^ and Mn^2+^. The calculation used an activity model that is similar to the Davies equation, based on the data of Morel and Hering, which is recommended to be applicable up to maximum ionic strength (I) of less four molal [30].

Table 2 summarizes the data of solubility products, complex stability constants, and acid dissociation constants used in the calculation. The stability constant of manganese gluconate was not available in the literature. However, it can be calculated using the polarographic measurement data that are available from the work of Bodini et al. [31], using the method of Heyrowskỳ and Lingane [32]. The details of manganese gluconate stability constants and equilibrium calculation methods are provided in the Supporting Information (SI–S1).

**Table 2:** Input Parameters used for equilibrium calculation of manganese and iron solution with NaHCO_3_, NaHS, NaOH and sodium gluconate.

Parameters	Value	Reference
• pK_a1a_ H_2_S	7.02	[28]
• pK_a2a_ H_2_S	12.87 & 19	[28]
• pK_a1a_ H_2_CO_3_	6.3	[33]
• pK_a2a_ H_2_CO_3_	10.32	[33]
• pK_a2a_ HGH	3.7	[15]
• pKspa ${\text{MnCO}}_{3}$/${\text{FeCO}}_{3}$	9.17 & 11.37/10.54	[30, 33]
• pkspa ${\text{MnS}}_{2}$/${\text{FeS}}_{2}$	3.7∗/7	[33]
• pKspa ${\text{Mn}\left(\text{OH}\right)}_{2}$/${\text{Fe}\left(\text{OH}\right)}_{2}$	-15.2∗∗/13.79	[33, 38]
Stability Constants log (βa)		
• $\text{Mn}{\left(\text{OH}\right)}^{+}$/$\text{ Fe}{\left(\text{OH}\right)}^{+}$	3.4/4.5	[33]
• $\text{Mn}{\left(\text{OH}\right)}_{2}^{\text{o}}$/$\text{Fe}{\left(\text{OH}\right)}_{2}^{\text{o}}$	5.8/7.4	[33]
• $\text{Mn}{\left(\text{OH}\right)}_{3}^{-}$/$\text{Fe}{\left(\text{OH}\right)}_{3}^{-}$	7.7/11	[33]
• $\text{Mn}{\left(\text{OH}\right)}_{4}^{=}$/$\text{Fe}{\left(\text{OH}\right)}_{4}^{=}$	7.7/8.9	[33]
• Stability Constants log (βa)		
• $\text{Fe}{\left({\text{HCO}}_{3}\right)}^{+}$	2.17	[36]
• $\text{Fe}{\left({\text{CO}}_{3}\right)}^{\text{o}}$	4.73	[36]
• $\text{Fe}\left(\text{II}\right)\left({\text{GH}}_{4}\right){\left(\text{OH}\right)}_{4}^{-2}$	17.8	[37]
• ${\text{E}}_{{\text{Mn}}^{2+}/\text{Mn}}^{\text{o}}$(volt SHE)	-1.431	[39]
• $\text{Mn}{\left({\text{HCO}}_{3}\right)}^{+}$	1.95	[36]
• $\text{Mn}{\left({\text{CO}}_{3}\right)}^{\text{o}}$	4.1	[36]
• $\text{Mn}\left(\text{II}\right){\left({\text{GH}}_{3}\right)}_{2}^{-2}$	11.0	this work
• $\text{Mn}\left(\text{III}\right){\left({\text{GH}}_{3}\right)}_{2}{\left(\text{OH}\right)}^{2-}$	42.1	this work
• $\text{Mn}\left(\text{IV}\right){\left({\text{GH}}_{3}\right)}_{2}{{\left(\text{OH}\right)}_{3}}^{3-}$	17.2	this work
• $\text{Fe}\left(\text{II}\right){\left({\text{GH}}_{4}\right)}^{+}$	10	[16]
• $\text{Fe}\left(\text{III}\right)\left({\text{GH}}_{4}\right){\left(\text{OH}\right)}_{3}^{-1}$	37.9	[37]
• $\text{Fe}\left(\text{III}\right)\left({\text{GH}}_{4}\right){\left(\text{OH}\right)}_{4}^{-2}$	37.2	[16]

∗ Based on reaction of ${\text{MS}}_{2\left(\text{s}\right)}+{\text{ H}}^{+}\;⇋\;{{\text{M}}^{2+}}_{\left(\text{aq}\right)}\;+\text{H}{{\text{S}}^{-}}_{\left(\text{aq}\right)}\;+{\text{S}}^{\text{o}}$.

∗∗ Based on reaction of ${\text{Mn}\left(\text{OH}\right)}_{2\left(\text{s}\right)}+\;2{\text{H}}^{+}\;⇋\text{ M}{{\text{n}}^{2+}}_{\left(\text{aq}\right)}\;+2{\text{H}}_{2}\text{O}$.

## 4 Results and discussion

### 4.1 Formation of elemental sulfur

**Figure 1:**
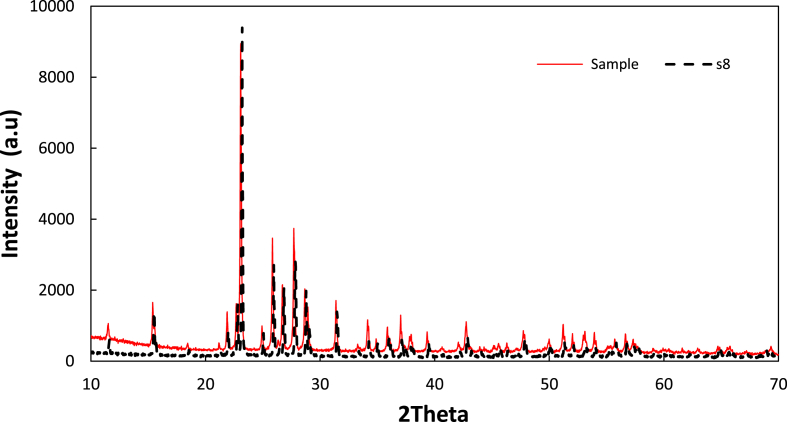
XRD spectra comparison between precipitate product compared to the pure elemental sulfur (S_8_). *Note*: Concentration of total manganese was 0.1 M, total gluconate was 1.2 M at pH of 13.

Elemental sulfur was produced in all of the manganese gluconate NaHS experiments performed. Figure 1 shows XRD spectrum of a precipitate solid obtained at a pH of 13.

It shows that the spectrum of the precipitate (red line) matched very well with the referenced spectrum of elemental sulfur S_8_ (black line). Although it has not been studied in detail, this experiment also showed the ease of manganese (II) to be oxidized by air since the NaHS oxidation into sulfur must occur due to the presence manganese of higher oxidation state. The change in oxidation state was indicated by the change in the color of the solution [31]. Used of re-aerated filtrate for fresh NaHS feed still resulted in elemental sulfur production. All of these established ones of the most important proves of the potential of the manganese gluconate as a sulfur recovery redox agent. A gravimmetric analysis indicated that the amount of sulfide recovered as elemental sulfur varied between 90 and 95% and increased with the pH.

### 4.2 Comparison of precipitation equilibrium calculation and experimental results

The oxidation of sulfide into elemental sulfur in a manganese gluconate LRSR system can be described by the following simplified desirable reactions (1) to (2). Reaction (1) is the sulfide oxidation by manganese (III) gluconate. Regeneration reaction of manganese (II) gluconate by oxygen back to manganese (III) gluconate is represented by reaction (2).

(1) $$4Mn\left(III\right){\left(GH_{3}\right)}_{2}{\left(OH\right)}^{2-}+2HS^{-}⇋4Mn\left(II\right){{\left(GH_{3}\right)}_{2}}^{-2}+2OH^{-}+1/4S_{8}+2H_{2}O$$

(2) $$4Mn\left(II\right){\left(GH_{3}\right)}_{2}^{-2}+O_{2}+2H_{2}O\Leftrightarrow 4Mn\left(III\right){\left(GH_{3}\right)}_{2}{\left(OH\right)}^{2-}$$

However if the manganese gluconates complex were not stable enough it may greatly decompose into manganese ions. These manganese ions will react with sulfide, carbonate, and hydroxide forming metal precipitates which will hinder the effectiveness of manganese to oxidize the sulfide. It is in this context that this work would like to evaluate the extent of the complex stability by performing precipitation experiments.

**Figure 2:**
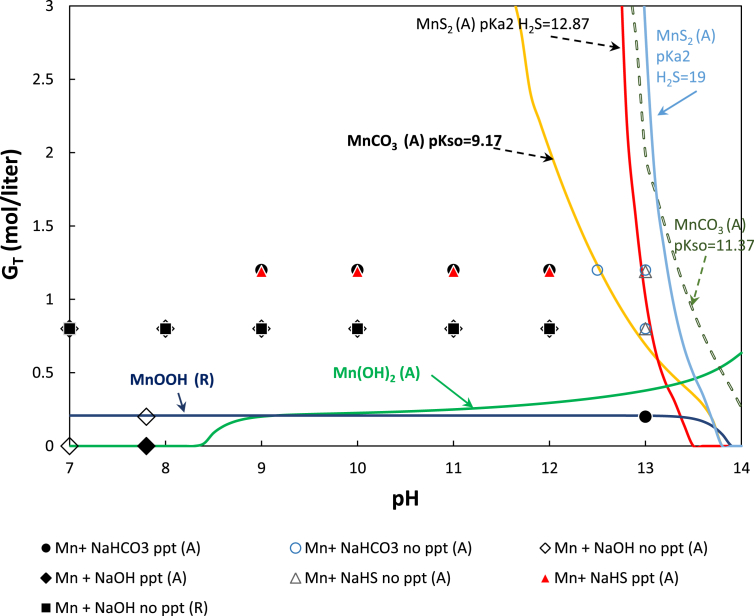
Solubility chart of manganese gluconate as function of pH and gluconate concentration. *Note*: Filled markers mean precipitation occured while unfilled markers mean unprecipitated (no salt precipitation). The experiment was conducted at concentration of total manganese, total carbonate, and total sulfide in the solutions were 0.1 M, 0.3 M, and 10 mM respectively.

The results of the equilibrium calculation and precipitation experiments for manganese systems are presented in Figure 2. Calculation results are given in the form of curves of [*G*_*T*_]_*sat*_ at different manganese salts as a function of pH. At a defined pH when the concentration of total gluconate is less than [*G*_*T*_]_*sat*_ for MnCO_3_, the solution should become oversaturated with manganese carbonate. The quantity of total ligands present is not enough to form a complex with sufficient amount manganese. This makes the remaining free manganese concentration in the system was higher than its solubility, i.e [Mn^2+^]_*sat*_. Hence from the thermodynamic aspect, the manganese carbonate should precipitate. Oppositely, in the region above the saturation curve, manganese precipitation should not take place. The same reasoning applies to the manganese gluconate NaHS and manganese gluconate only system.

As shown in Figure 2, at pH of 9 and 10, significant precipitation occurred rapidly after the mixing of 0.1 M of Mn gluconate in 1 M of sodium gluconate solution with 0.3 M of NaHCO_3_ solution. Upon verification with XRD, the brown precipitates were identified as synthetic rhodhochrosite manganese carbonate. The same phenomenon was observed at pH of 7 but occured in a longer period (*ca*. one day). The process still even took place when the concentration of total manganese and total carbonate were reduced to 0.03 M and 0.01 M, respectively, or when the pH was increased to 10 and 12. Solutions that were stable after four weeks of observation were obtained when the pH was increased to 13. At the same pH precipitation was again found when the ligand to metal ratio was decreased to 0.2. All of these results were in agreement with the prediction of the equilibrium calculation of the manganese carbonate system when the solubility product used was according to the data of Sternbeck [29]. Solubility data from Jensen et al. [30] gave a higher pH requirement for preventing the precipitation of manganese carbonate. Figure 2 also shows that manganese (II) hydroxide saturation was started at pH of 8.4 when gluconate was not added. However, in the experiment, it was precipitated at pH of slightly lower than 8. When total gluconate to total manganese ratio was 8–12, the solution was stable against hydroxide precipitation at all range of tested pHs.

In agreement with the equilibrium calculation at manganese to gluconate ratio of 1.2, there was significant manganese-sulfide precipitation at all pH ranges, except when the pH is more than 12.5. The weight of dried precipitates obtained within pH range of 7–9 was considerably higher than the amount of sulfur content of the feed. The similarity between the XRD spectra of the precipitate samples and the sulfur standard (S_8_) was less than 50%. Furthermore, the color of precipitates shifted from black to brown and finally yellow when the pH was increased from 7 to 12.5. It is concluded that manganese did precipitate significantly in the higher pH region (pH > 9). When the pH increased, the amount of precipitation became less. At pH of 13, the XRD analysis indicated that the probability that the sample was elemental sulfur reached 95%. This result was comparable to the weight reduction of precipitate samples obtained from oxidation with Fe-EDTA at pH of 8–9.

In addition to the model for absorption process, Figure 2 presents the saturation curve of Mn(III)OOH that was obtained by air bubbled regeneration. It shows that the gluconate addition requirement for preventing MnOOH saturation is relatively not high.

Based on this experiment it is concluded that precipitation of manganese can indeed be avoided or minimized by pH adjustment, and therefore from this aspect, manganese gluconate solution has the potential to be used as a redox agent for LRSR but, in contrast to Fe-NTA, manganese gluconate requires a higher pH for stability not to be precipitated.

### 4.3 Comparison of manganese and iron system

**Figure 3:**
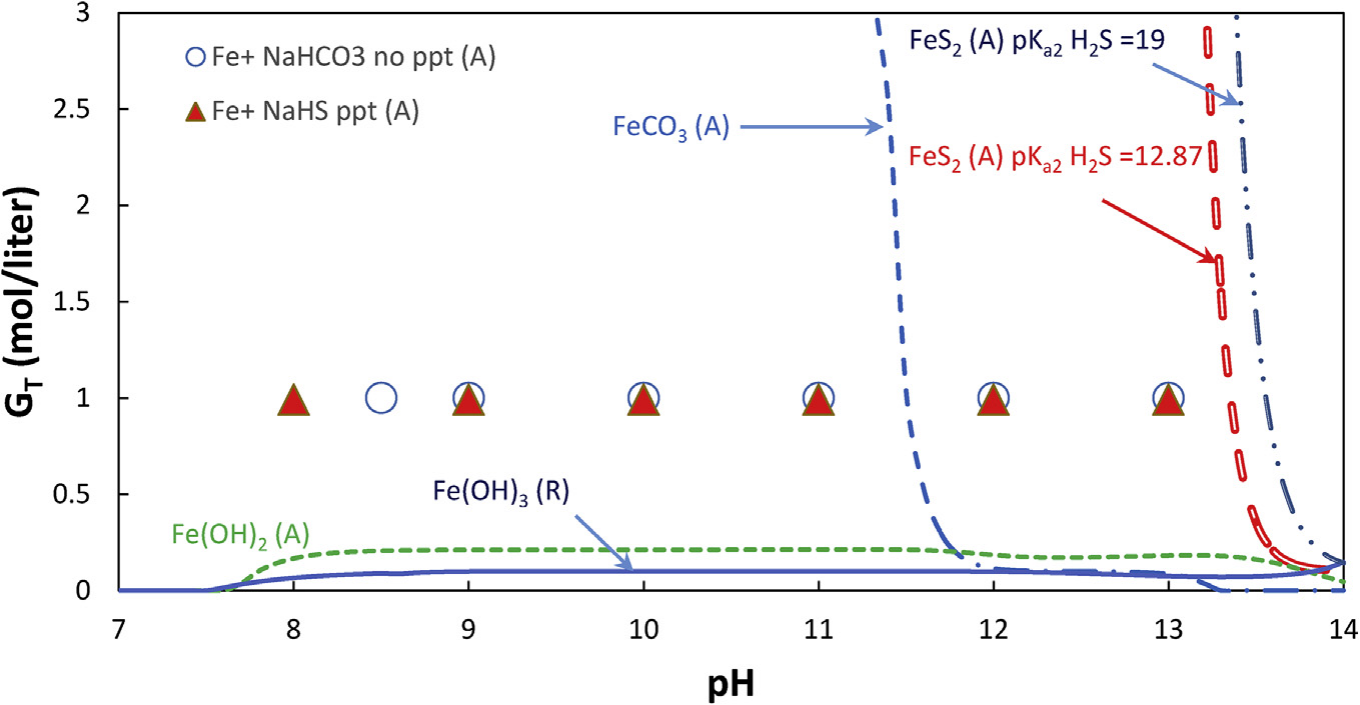
Solubility chart of iron gluconate as function of pH and gluconate concentration. *Note*: Filled markers mean precipitation occured while unfilled markers mean unprecipitated (no salt precipitation). The experiment was conducted at concentration of total iron, total carbonate, and total sulfide in the solutions were 0.1 M, 0.3 M, and 10 mM respectively.

Figure 3 compares the equilibrium calculation with experimental observation for iron gluconate sodium bicarbonate, iron gluconate NaHS and iron-gluconate solutions. Several essential differences between manganese and iron system can be pointed out. Manganese carbonate precipitation occured at pH 8–11 while no precipitation of iron (II) –gluconate-bicarbonate system was found. Since the equilibrium model indicated that the iron carbonate solution in this region was oversaturated, the stability of iron carbonate may be caused by slow kinetics of precipitation. The second difference between the two systems was that manganese sulfide precipitated at a slower rate than iron sulfide. In the case of iron gluconate-NaHS-sodium gluconate system, the precipitated product has a black color, indicating that the precipitate mostly contains stable iron sulfide. Deshpande [26] mentioned that the black particles could be transformed into elemental sulfur upon air oxidation but, in our work, the black particles remained black after overnight bubbling by air.

The rapid precipitation of iron (II) sulfide and manganese (II) carbonate, and the slower precipitation of iron (II) carbonate and manganese sulfide may be related to the Hard Soft Acid-Base (HSAB) principle [40].

Carbonates are stronger base ion than sulfide ions. Oxygen as the electron donor in the carbonate ion is more electronegative than sulfide. Hence compared to sulfide in a ligand system that anions have to share their electron to the metal ions, oxygen is easier to share the electron with the metal. On the other hand, Mn^2+^ ions have stronger acidity properties than Fe^2+^. Compared with iron, therefore, manganese will be easier to develop electrostatic attraction with the strong base carbonate ions, forming ionic pair cluster which smoothens the way for nucleation and growth of manganese carbonate precipitate. The reverse situation occured to explain a rapid precipitation of iron (II) sulfide. Because of the formation of sulfide precipitate, the iron gluconate has less possibility to be applied in the LRSR process.

### 4.4 Oxidative degradation resistant comparison

It was observed that the iron NTA started to form visible brown precipitate after two weeks. In contrast, both of the manganese gluconate solutions did not form any precipitate even after four weeks of observation. Since the metals in all of the solutions were soluble at the beginning and the first days of the experiments, the formation of precipitate from NTA was a sign that a significant quantity of the ligand had been degraded into non-ligand substances. These indicated that the oxidative degradation rate of gluconate in manganese gluconate was lower than that of NTA in iron gluconate. Since it was reported that NTA was more resistant to degradation than EDTA, this also meant that gluconate was better than EDTA in this respect.

## 5 Conclusions

Manganese gluconate complex was not as stable as conventional complex when operated in an LRSR system at relatively low pH. However, operated at pH higher than 11, initial assesment has shown its potential as a new complex for Liquid Redox Sulfur Recovery (LRSR). In this condition, the complex was stable against metal precipitation and was capable of producing elemental sulfur with 90–95% yield, quite rapidly. Furthermore, experiments also suggested that gluconate ligand was more resistance toward oxidative degradation than NTA and EDTA. Combined with the lower price of gluconate, the complex could be a promising LRSR agent and therefore deserved more detailed investigations.

## Declarations

### Author contribution statement

Tirto Prakoso: Analyzed and interpreted the data.

Andreas Widodo: Conceived and designed the experiments; Performed the experiments; Wrote the paper.

Antonius Indarto: Conceived and designed the experiments; Wrote the paper.

Rina Mariyana: Performed the experiments; Analyzed and interpreted the data.

Aditya Farhan Arif: Analyzed and interpreted the data; Wrote the paper.

Tri Partono Adhi: Conceived and designed the experiments; Analyzed and interpreted the data.

Tatang Hernas Soerawidjaja: Conceived and designed the experiments.

### Funding statement

The research was supported by PT. Rekayasa Industri, Engineering & Construction, Indonesia. This research is also partially funded by the Indonesian 10.13039/501100009509Ministry of Research, Technology and Higher Education under the World Class University (10.13039/100007176WCU) Program managed by Institut Teknologi Bandung.

### Competing interest statement

The authors declare no conflict of interest.

### Additional information

No additional information is available for this paper.
