# Supplementary material for: Manganese gluconate, A greener and more degradation resistant agent for H2S oxidation using liquid redox sulfur recovery process
Source: Heliyon. 2020 Feb 10;6(2):e03358. doi: 10.1016/j.heliyon.2020.e03358 (PMC8045144; doi:10.1016/j.heliyon.2020.e03358)
Supplement: Supporting Information [file mmc1.docx]

**Supporting information for: Manganese Gluconate, A Greener and More Degradation Resistant Agent for H_2_S Oxidation Using Liquid Redox Sulfur Recovery Process**

Tirto Prakoso^1^, Andreas Widodo^2,^*, Antonius Indarto^1^, Rina Mariyana^3^, Aditya Farhan Arif^3^, Tri Partono Adhi^1^, Tatang Hernas Soerawidjaja^1^

1. Department of Chemical Engineering, Institut Teknologi Bandung, Labtek X, Kampus ITB, Jalan Ganesha 10, Bandung 40132, Indonesia
2. PT. Energy Management Indonesia (EMI), Jl. Pancoran Indah I No.52, Jakarta, Indonesia
3. PT. Rekayasa Industri (REKIND), Jl. Kalibata Timur I no 36, Jakarta, Indonesia

*** Corresponding author. Tel.: +62-811-917-601 (Andreas Widodo).**

a.widodo.wr@gmail.com (Andreas Widodo)

**Number of pages : 7**

**Number of Figures : 0**

**Number of description : 2**

**Equilibrium Calculation Formulation** **SI-S1.**

**Calculation of the Stability Constant of Manganese Gluconates**

Stability constants of complexes can be calculated from polarographic measurement data [27]. Bodini et al., measured half wave potentials ($E_{1/2}$) of manganese gluconate solution as a function of hydroxide ion ([OH^-^]), and gluconate ion concentration ([(GH_4_)^-^) [31]. The measurement was carried out for different changes of oxidation states. These data has been used in this work to determine stability constant of manganese (II), (III) and (IV) gluconate complexes. Calculation of manganese (II) gluconate stability constant will be described as an example. The same principle was used for estimating manganese (III) and (IV) gluconates stability constant.

The changes in oxidation state of manganese from (II) to (0), was effected by the reduction of free manganese (II) ions ($\mathrm{Mn}^{2+})$to manganese metal taking place at the DME (dropping mercury electrode) surface described by reaction 1. Equation 2 is the Nernst equation for this reaction.

$\mathrm{Mn}^{2+}+2e\Longleftrightarrow Mn\left( 0 \right) E_{{{Mn}^{2+}}/{Mn}}^{o}=-1.431 V SCE {(standard Calomel Electrode)}$ [39] (1)

$E=E_{{\mathrm{Mn}^{2+}}/\mathrm{Mn}}^{o}-\frac{0.059}{2}\log\left( \frac{1}{\left\{ \mathrm{Mn}^{+2} \right\}_{s}} \right)$ (2)

E is redox potential, $E_{{\mathrm{Mn}^{2+}}/\mathrm{Mn}}^{o}$ is redox potential for the reduction reaction 2 at standard condition in SCE, and $\left\{ \mathrm{Mn}^{+2} \right\}_{s}$ is activity of $\mathrm{Mn}^{2+}$at the surface of DME. The unit of $E_{{\mathrm{Mn}^{2+}}/\mathrm{Mn}}^{o}$ is given in SCE as the unit of ($E_{1/2}$) in Bodini et al.,’s work is also in SCE.

The activity of free manganese (II) ion from equation 3 can then be substituted to equation 2. Equation 3 is the equilibrium relation of manganese (II) gluconate formation in reaction (4). Subscript s indicates surface of DME, curly bracket represent activity, $GH_{3}$ is gluconate ion with two hydrogen deprotonized and $\beta_{a\mathrm{Mn}\left( \mathrm{II} \right){(GH_{3})_{2}}^{-2}}$ is the activity based stability constant of manganese (II) gluconate complex.

$\left\{ \mathrm{Mn}^{+2} \right\}_{s}=\frac{\left\{ \mathrm{Mn}\left( \mathrm{II} \right){(GH_{3})_{2}}^{-2} \right\}_{s}}{\left\{ \left( GH_{4} \right)^{-} \right\}_{s}^{2}\left\{ \mathrm{OH}^{-} \right\}_{s}^{2}\beta_{a\mathrm{Mn}\left( \mathrm{II} \right){(GH_{3})_{2}}^{-2}}}$ (3)

$\mathrm{Mn}^{2+}+2\left( GH_{4} \right)^{-}+2\mathrm{OH}^{-}\leftrightharpoons Mn\left( \mathrm{II} \right){(GH_{3})_{2}}^{-2}+2H_{2}O$ (4)

By replacing the surface activity terms in the Nerst equation with bulk concentration, equation 5 can be established. This equation relates $E_{1/2}$ to hydroxyde concentration, with A is a constant defined by equation 6. In the latter equation the activity terms in the Nernst equation have been converted to bulk concentration and activity coeficients. Concentration in the bulk is indicated by the subscript o.

$E_{1/2}=A-0.059 log\left[ \mathrm{OH}^{-} \right]_{O}$ (5)

where

${A=E}_{{\mathrm{Mn}^{2+}}/\mathrm{Mn}}^{o}-\frac{0.059}{2}\log\left( \frac{\left[ \left( GH_{4} \right)^{-} \right]_{o}^{2}}{\frac{1}{2}\left( \left[ \mathrm{Mn}\left( \mathrm{II} \right){(GH_{3})_{2}}^{-2} \right]_{o} \right)}\frac{\gamma_{\left( GH_{4} \right)_{o}^{-}}^{2}\gamma_{OH_{o}^{-}}^{2}}{\gamma_{\mathrm{Mn}\left( \mathrm{II} \right){{(GH_{3})_{2}}^{-2}}_{o}}} \right)-\frac{0.059}{2}\log\left( \beta_{a\mathrm{Mn}\left( \mathrm{II} \right){(GH_{3})_{2}}^{-2}} \right)$ (6)

The conversion of activities of manganese gluconate to bulk concentration and activity coefficient was obtained by the use of the definition of half wave potential and diffusional current (i) as described by equation 7 and 8 with k is a constant.^28^

$i=k\left( \left\{ \mathrm{Mn}\left( \mathrm{II} \right){(GH_{3})_{2}}^{-2} \right\}_{o}-\left\{ \mathrm{Mn}\left( \mathrm{II} \right){(GH_{3})_{2}}^{-2} \right\}_{s} \right)$ (7)

when the second term in equation is zero the current becomes the limiting current ($i_{\mathrm{ld}}$) as given in equation 8.

$i_{\mathrm{ld}}=k \left\{ \mathrm{Mn}\left( \mathrm{II} \right){(GH_{3})_{2}}^{-2} \right\}_{o}$ (8)

with E_1/2_ being the potential electrode when $i={\frac{1}{2}i}_{\mathrm{ld}}$, equation 7 and 8 can be combined to give equation 9 in which the right hand term can be replaced with concentration and activity coefficient at the bulk.

$\left\{ \mathrm{Mn}\left( \mathrm{II} \right){(GH_{3})_{2}}^{-2} \right\}_{s}={\frac{1}{2}\left\{ \mathrm{Mn}\left( \mathrm{II} \right){(GH_{3})_{2}}^{-2} \right\}}_{o}$ (9)

As for hydroxide and gluconate ions, it was approximated by carrying out the respective surface mass balance and assuming uniformity of concentration at the beginning of the process. Equation 10 relates hydroxide concentration at surface when the half wave potential is attained ($\left[ \mathrm{OH}^{-} \right]_{s{,@E}_{1/2}})$ to its initial concentration ${(\left[ \mathrm{OH}^{-} \right]}_{s, initial}).$

$\left[ \mathrm{OH}^{-} \right]_{s{,@E}_{1/2}}=\left[ \mathrm{OH}^{-} \right]_{s, initial}+\Delta\left[ \mathrm{OH}^{-} \right]\approx\left[ \mathrm{OH}^{-} \right]_{o}+\Delta\left[ \mathrm{OH}^{-} \right]$ (10)

$\Delta\left[ {OH}^{-} \right]$ the amount of hydroxide ions generated by the reaction within the period from the start of the reaction until the half wave potential is attained. Referring to reaction 4, the hydroxide generated will be the same as $\mathrm{Mn}\left( \mathrm{II} \right){(GH_{3})_{2}}^{-2}$ shifted to the left as $\mathrm{Mn}^{2+}$ converted to Mn(0). Since in Bodini et al.’s experiment the concentration of manganese gluconate (5mM) is significantly smaller than hydroxide concentration (0.3 M), equation 10 becomes equation 11.

$\left[ \mathrm{OH}^{-} \right]_{s{,@E}_{1/2}}\approx\left[ \mathrm{OH}^{-} \right]_{o}$ (11)

The same approach was applied to the gluconate concentration.

Equation 12 is the activity model used for calculating the coefficient of activities ($\gamma_{j})$ for component j with a charge of z_j_. The used such model makes equation 5 become a linear equation that relates $E_{1/2}$ and $-log\left[ \mathrm{OH}^{-} \right]_{O}$. Fitting equation 5 with $E_{{\mathrm{Mn}^{2+}}/\mathrm{Mn}}^{o}=-1.431 Volt vs SCE$ into the Bodini et al.‘s data, gives $\beta_{a\mathrm{Mn}\left( \mathrm{II} \right){(GH_{3})_{2}}^{-2}}=3.8 x{10}^{10}$.

$\log\gamma_{j}=-0.509 {z_{j}}^{2}\left( \frac{\sqrt{I}}{1+1.49\sqrt{I}}-0.11I \right)$ (12)

The same calculation has been done for the data of half wave potential as function of gluconate concentration at constant hydroxide concentration, giving an average of stability constant of $\beta_{a\mathrm{Mn}\left( \mathrm{II} \right){(GH_{3})_{2}}^{-2}} equals 1.1 x{10}^{11}$. The concentration based stability constant ($\beta_{c\mathrm{Mn}\left( \mathrm{II} \right){(GH_{3})_{2}}^{-2}}$) at different ionic strengths can then be estimated from $\beta_{a\mathrm{Mn}\left( \mathrm{II} \right){(GH_{3})_{2}}^{-2}}$ using equation 13.

$\beta_{c\mathrm{Mn}\left( \mathrm{II} \right){(GH_{3})_{2}}^{-2}}=\beta_{\mathrm{act}\mathrm{Mn}\left( \mathrm{II} \right){(GH_{3})_{2}}^{-2}}\frac{\gamma_{\mathrm{Mn}^{+2}}\gamma_{OH^{-}}^{2}\gamma_{\left( GH_{4} \right)^{-}}^{2}}{\gamma_{\mathrm{Mn}\left( \mathrm{II} \right){(GH_{3})_{2}}^{-2}}}$ (13)

**Equilibrium Calculation Formulation SI-S2.**

**Equilibrium Calculation for Manganese Gluconate Stability Against Precipitation**

This section describes the calculation of total gluconate concentration at saturation condition needed to prevent precipitation ${(\left[ G_{T} \right]}_{sat}$) that have been carried out, using manganese gluconate-sodium gluconate-NaHCO_3_ system as an example. For the other systems $\left[ G_{T} \right]_{sat}$have been obtained using the same method. The calculation began with assignment of a guessed value for gluconate ion concentration at saturation $\left[ {(GH_{4})}^{-} \right]_{sat}$ followed by construction of charge balance given in equation 14 or 15 to determine concentration of sodium ions. This balance assumed that $\left[ \mathrm{Mn}^{2+} \right]_{sat}+\left[ \mathrm{Mn}^{3+} \right]_{sat}+ \left[ H^{+} \right] \ll\left[ {Na}^{+} \right],$and that most of the manganese exists in the form of Mn(III) gluconate. As a consequence of the latter, $Mn(III)(GH_{3})_{2}(OH)^{2-}$ concentration could be approximated by the total manganese concentration $\left[ (Mn)_{T} \right]$. This simplification was taken since manganese(II) gluconate complex has a much greater stability constant compared to all other manganese complexes. Further, hydroxide complexes could be considered to be significant only when pH is more than 13, and therefore in this context they were neglected. Finally when for pH between 7 and 12, most carbonates would be in the form of bicarbonate ions, while for higher pH, in the form of carbonate ions. Thus, the bicarbonate or carbonate ions concentration in its respective pH could be approached with total carbonate concentration $(C_{T})$

$\left[ \mathrm{Na}^{+} \right]\approx\left[ \mathrm{OH}^{-} \right]+2\left[ (Mn)_{T} \right]+\left[ \left( GH_{4} \right)^{-} \right]+C_{T}$ pH= 7 -10 (14)

$\left[ \mathrm{Na}^{+} \right]\approx\left[ \mathrm{OH}^{-} \right]+2\left[ (Mn)_{T} \right]+\left[ \left( GH_{4} \right)^{-} \right]+2C_{T}$ pH= 10 -13 (15)

Since all dominant components had become known, the ionic strength (I) was then estimated using equation 16. The process continued with calculation of coefficient of activity for components with different electrical charges using equation 12.

$I=\frac{1}{2}\sum_{j} \left[ j \right]z_{j}^{2}\approx\frac{1}{2}(\left[ \mathrm{Na}^{+} \right]+\left[ \mathrm{OH}^{-} \right]+4\left[ (Mn)_{T} \right]+\left[ \left( GH_{4} \right)^{-} \right]+C_{T})$ (16)

The next step was determining all concentration-based stability constants, dissociation constants, and solubility products (β_c_, K_ac_ and K_spc_) using corresponding activity based constants (β_a_, K_aa_ and K_spa_) data such as in equations 13 and 17.

$K_{spcMnCO3}=\frac{K_{spactMnCO3}}{\gamma_{{Mn}^{+2}}\gamma_{{CO3}^{=}}}$ (17)

Afterward, carbonate mass balance, and equilibrium relation for the first ($K_{a1cH_{2}\mathrm{CO}_{3}})$and second ($K_{a2cH_{2}\mathrm{CO}_{3}})$dissociation of carbonic acid were used to find out the concentration of bicarbonate and carbonate ions. The balance is given in 18, while equation 19, and 20 give the results.

$C_{T}=\left[ {H\mathrm{CO}_{3}}^{-} \right]+\left[ {\mathrm{CO}_{3}}^{=} \right]+\left[ H_{2}\mathrm{CO}_{3} \right]+\mathrm{MnCO}_{3}^{0}+\mathrm{MnHCO}_{3}^{+}$ `(18)

$\left[ {\mathrm{HCO}_{3}}^{-} \right]=\frac{\left( C_{T}-\beta_{cMnCO3}K_{spcMnco3}+\beta_{cMnCO3}\frac{K_{spcMnco3}}{K_{a2cH_{2}\mathrm{CO}_{3}}}\left[ H^{+} \right] \right)}{\alpha_{T}}$ (19)

$\left[ {\mathrm{CO}_{3}}^{=} \right]=\frac{K_{a2cH_{2}\mathrm{CO}_{3}}\left[ {\mathrm{HCO}_{3}}^{-} \right]}{\left[ H^{+} \right]}$ (20)

where

$\alpha_{c}=\left( 1+\frac{\left[ H^{+} \right]}{K_{a{1cH}_{2}{CO}_{3}}}+\frac{K_{a{2cH}_{2}{CO}_{3}}}{\left[ H^{+} \right]} \right)$ (21)

Application of solubility product relation gave equation 22 which specifies the concentration of free manganese (II) ions in a solution saturated with manganese carbonate, $\left[ \mathrm{Mn}^{2+} \right]_{\mathrm{sat}}$.

$\left[ \mathrm{Mn}^{2+} \right]_{\mathrm{sat}}=\frac{K_{spc,Mn\mathrm{CO}_{3}}}{\left[ {\mathrm{CO}_{3}}^{=} \right]}$ (22)

Reaction 23, and equilibrium relation in equation 24, were employed to generate equation 25 i.e., the relation between concentration of free manganese (III) and free manganese (II) ions.

$\mathrm{Mn}^{3+}+e^{-}\leftrightharpoons\mathrm{Mn}^{2+} E^{o}=+1.5 V$ SHE (Standard Hydrogen Electrode)^38^ (23)

$K=exp\left( \frac{\mathrm{nF}E^{o}}{\mathrm{RT}} \right)$ (24)

$\frac{\left[ \mathrm{Mn}^{3+} \right]_{\mathrm{sat}}}{\left[ \mathrm{Mn}^{2+} \right]_{\mathrm{sat}}}=\frac{2.74 {x 10}^{-24}}{{10}^{-pE}\gamma_{\mathrm{Mn}^{+3}}}\gamma_{\mathrm{Mn}^{+2}}$ (25)

with $pE=\frac{2.303RT}{F}E$ (26)

K is equilibrium constant while E is redox potential. Once the above free ion concentrations have been known, concentration of hydroxide and carbonate or bicarbonate complexes could be calculated by equation 27-29.

$\left[ \mathrm{Mn}\left( \mathrm{OH} \right)_{j}^{2-i} \right]=\beta_{\mathrm{Mn}\left( \mathrm{OH} \right)_{j}^{2-j}c}\left[ \mathrm{Mn}^{2+} \right]_{\mathrm{sat}}\left[ \mathrm{OH} \right]^{i}$ for j =1 to 4 (27)

$\left[ \mathrm{MnH}\mathrm{CO}_{3}^{+} \right]=\beta_{\mathrm{MnH}\mathrm{CO}_{3}^{+}c}\left[ \mathrm{Mn}^{2+} \right]_{\mathrm{sat}}\left[ {H\mathrm{CO}_{3}}^{-} \right]$ (28)

$\left[ \mathrm{Mn}\mathrm{CO}_{3}^{o} \right]=\beta_{\mathrm{Mn}\mathrm{CO}_{3}^{o}c}\left[ \mathrm{Mn}^{2+} \right]_{\mathrm{sat}}\left[ {H\mathrm{CO}_{3}}^{-} \right]$ (29)

In contrast to this, up to this point manganese gluconate complexes concentrations was only obtained in terms of $\left[ {(GH_{4})}^{-} \right]_{sat}$ using equation 30.

$\left[ \mathrm{Mn}\left( n \right){(GH_{3})_{2}}^{-2}(OH)^{a-} \right]=\beta_{c,Mn\left( n \right){(GH_{3})_{2}}^{-2}(OH)^{a-}}\left[ \mathrm{Mn}^{n+} \right]_{\mathrm{sat}}\left[ \left( GH_{4} \right)^{-} \right]^{2}\left[ \mathrm{OH}^{-} \right]^{b}/\left[ e^{-} \right]^{c}$ (30)

In equation 30, b and c is the coefficient of reaction of hydroxide ion and electron $e^{-}$ respectively in reaction 4 for manganese (II), and in reaction 31-32 for manganese (III) and (IV) respectively. The oxidation state of manganese is represented by n, whereas the number of hydroxide in the complex by *a*. Equation 15, 18 and 20 to 23 can then be subtituted to manganese mass balance in equation 33 to determine calculated $\left[ \left( GH_{4} \right)^{-} \right].$This $\left[ {(GH_{4})}^{-} \right]_{sat}$ concentration was then compared to the guessed $\left[ {(GH_{4})}^{-} \right]_{sat}$. The process was repeated until the difference between guessed and calculated $\left[ {(GH_{4})}^{-} \right]_{sat}$ was acceptable. Once this is reached, $\left[ {(GH_{4})}^{-} \right]_{sat}$can be used to obtained $\left[ G_{T} \right]_{sat}$ from equation 34.

$\mathrm{Mn}^{3+}+2\left( GH_{4} \right)^{-}+3\mathrm{OH}^{-}\leftrightharpoons Mn(III)(GH_{3})_{2}(OH)^{2-}+2H_{2}O$ (31)

$\mathrm{Mn}^{2+}+2\left( GH_{4} \right)^{-}+5\mathrm{OH}^{-}\leftrightharpoons Mn(IV)(GH_{3})_{2}{\left( \mathrm{OH} \right)_{3}}^{3-}+{2e}^{-}+2H_{2}O$ (32)

$\left[ \mathrm{Mn}_{T} \right]=\left[ \mathrm{Mn}^{2+} \right]_{sat}+\left[ \mathrm{Mn}^{3+} \right]_{sat}+\sum\left[ MnGH \right]_{\mathrm{sat}}+\left[ \mathrm{MnH}\mathrm{CO}_{3}^{+} \right]_{sat}+\left[ \mathrm{Mn}\mathrm{CO}_{3}^{o} \right]_{sat}+\sum\left[ \mathrm{Mn}{(OH)}_{x}^{y} \right]_{\mathrm{sat}}$ (33)

$\sum\left[ MnGH \right]_{\mathrm{sat}}$ and $\sum\left[ \mathrm{Mn}{(OH)}_{x}^{y} \right]_{\mathrm{sat}}$ are the sum concentration of all manganese gluconate and manganese hydroxide complexes, respectively.

$\left[ G_{T} \right]_{sat}=\left[ {(GH_{4})}^{-} \right]_{sat}\left( 1+\frac{\left[ H^{+} \right]}{K_{agh}} \right)+2\sum\left[ MnGH \right]_{\mathrm{sat}}$ (34)
